# A Novel Integrated Active Herbal Formulation Ameliorates Dry Eye Syndrome by Inhibiting Inflammation and Oxidative Stress and Enhancing Glycosylated Phosphoproteins in Rats

**DOI:** 10.3390/ph13100295

**Published:** 2020-10-07

**Authors:** Omer Ersin Muz, Cemal Orhan, Fusun Erten, Mehmet Tuzcu, Ibrahim Hanifi Ozercan, Prafull Singh, Abhijeet Morde, Muralidhara Padigaru, Deshanie Rai, Kazim Sahin

**Affiliations:** 1 Department of Ophthalmology, Eskisehir Yunus Emre State Hospital, Eskisehir 26190, Turkey; ersinmuz@gmail.com; 2 Department of Animal Nutrition, Veterinary Medicine, Firat University, Elazig 23119, Turkey; corhan@firat.edu.tr; 3 Department of Biology, Faculty of Science, Firat University, Elazig 23119, Turkey; fusun_87@yahoo.com (F.E.); mtuzcu@firat.edu.tr (M.T.); 4 Department of Pathology, Faculty of Medicine, Firat University, Elazig 23119, Turkey; ozercanih@firat.edu.tr; 5 OmniActive Health Technologies, Phoenix House, T- 8, A Wing 462 Senapati Bapat Marg, Lower Parel, Mumbai 400 013, India; s.prafull@omniactives.com (P.S.); a.morde@omniactives.com (A.M.); m.padigaru@omniactives.com (M.P.); d.rai@omniactives.com (D.R.)

**Keywords:** inflammation, oxidative stress, lutein, curcumin, vitamin D3, dry eye syndrome

## Abstract

Dry eye syndrome (DES) is a chronic condition of the eye with insufficient production of tears leading to inadequate lubrication of eyes. Symptoms of DES are associated with discomfort and redness of the eye, blurred vision, and tear film instability which leads to the damaged ocular surface. Inflammation and oxidative stress play a significant role in the pathogenesis of the disease. In this study, the protective effect of different doses (100 or 200 mg/kg) of a novel multi-component oral formulation of lutein/zeaxanthin, curcumin, and vitamin D3 (LCD) was evaluated using a rat model with benzalkonium chloride (BAC)-induced dry eye syndrome. The formulation was administered orally to rats for 4 weeks. We observed a significant improvement in tear volume, tear breakup time, tear film integrity, and reduction in overall inflammation in rats fed with the LCD at dose 200 mg/kg performing better than 100 mg/kg. Furthermore, the formulation helped in lowering oxidative stress by increasing antioxidant levels and restored protective tear protein levels including MUC1, MUC4, and MUC5AC with 200 mg of LCD having the most significant effect. The results strongly suggest that the combination of lutein/zeaxanthin, curcumin, and vitamin-D3 is effective in alleviating the symptoms of dry eye condition with a multi-modal mechanism of action.

## 1. Introduction

Dry eye syndrome (DES) is a multifactorial disease of the tear film and ocular surface of the eye leading to reduced tear production, symptoms of discomfort, and visual disturbance with potential damage to the ocular surface. DES is associated with tear film instability, the increased osmolarity of tear, and ocular surface inflammation [1]. The aged population and women are at significant risk of contracting DES and the disease prevalence ranges widely from 5 to 50% across the globe with a significant burden on quality of life [2,3]. The National Health and Wellness Survey 2013 reported a prevalence rate of 6.8% in the United States with 16.4 million adult population affected by DES [4]. Increased use of light-emitting diode displays such as cell phones, flashlights, computers, has further increased incidences of various symptoms associated with DES [5,6].

The pathophysiology of DES includes loss of tear film, tear hyperosmolarity, oxidative stress, and inflammation of ocular surface that result in a vicious cycle of ocular surface damage and inflammation [7]. Inflammation mediated by pro-inflammatory cytokines such as tumor necrosis factor-alpha (TNF-α), interleukin (IL)-6, and IL-8 is high in tears of dry eye patients. These cytokines inhibit the release of neurotransmitters, and the activity of sensory nerves on the ocular surface thus weakens the secretion of lacrimal glands. Pro-inflammatory pathways including nuclear factor-kappa B (NF-κB) and mitogen-activated protein kinase (MAPK) pathways are also implicated in DES [8,9,10,11]. Oxidative stress plays an important role in inducing damage to the ocular surface in DES with increased levels of malondialdehyde (MDA), lipid peroxidation markers, and decreased antioxidants such as superoxide dismutase (SOD), catalase, and glutathione peroxidase (GSH-Px) in the tear film and ocular surface of dry eye patients [12].

The first line of treatment for DES consists of the use of artificial tears followed by topical corticosteroids which usually improve the symptoms of the disease but are associated with adverse effects over long term use [13]. Hence, there has been a lot of interest in exploring the effectiveness of natural ingredients that can be administered orally avoiding frequent use and adverse events associated with long-term use of eyedrops. In this regard, several nutraceuticals have been explored such as n-3 long-chain fatty acids [14,15], carotenoids [16,17], anthocyanins [18,19,20], and antioxidants [21] in the prevention and treatment of DES [22].

In the present study, we used an optimized marigold extract as a source of lutein and zeaxanthin at a 5:1 ratio as occur in fruits and vegetables. Both lutein and zeaxanthin are xanthophyll carotenoids found in high concentrations in the macula of the eye which absorb high energy blue light and protect the retina from phototoxicity [23]. These ingredients also act as antioxidants and scavenge free radicals. Thus long-term supplementation of these natural ingredients is associated with protection against eye conditions such as age-related macular degeneration [24]. Furthermore, lutein and zeaxanthin are known to inhibit inflammatory pathways including lipopolysaccharide-induced secretion of IL-8 [17] and hyper-osmotically induced secretion of IL-6 in corneal epithelial cells [16].

Curcumin, a yellow-colored polyphenol from the plant *Curcuma longa*, known for anti-inflammatory and antioxidant properties, was used as another ingredient in the present formulation. Curcumin is extensively used for treatment in multiple disease conditions in Indian traditional medicine. Studies have demonstrated the beneficial effects of curcumin in multiple anterior segment eye diseases such as corneal disease, dry eye condition, conjunctivitis, anterior uveitis, cataracts, and glaucoma [25]. Curcumin inhibits pro-inflammatory cytokines, IL-4 and IL-5, expression in the conjunctiva in mice [26]. A study by Chen et al. showed that curcumin to be protective against hyper-osmotically induced IL-1β upregulation in corneal epithelial cells through p38 MAPK/NF-κB pathways [27].

Vitamin D is a multifunctional nutrient that is obtained from dietary consumption or produced by the skin following exposure to sunlight. It has been widely studied for its immune-modulatory properties such as suppression of lymphocyte proliferation, cytokine and chemokine expression, and antigen-presenting cell differentiation [28,29]. Further, vitamin D significantly decreased the expression of pro-inflammatory mediators in HCECs under hyperosmotic stress, probably through inhibiting NF-κB activation [20]. More importantly, low serum vitamin D levels are found to be associated with DES, and tear secretion and tear breakup time (TBUT) were positively correlated with serum vitamin D concentrations [21]. Furthermore, vitamin D receptor is found in the corneal epithelium, endothelium, and retinal pigmentary epithelium [30] and also enhances corneal epithelial barrier function [31,32]. From the available studies, we selected lutein/zeaxanthin, curcumin, and vitamin D3 (LCD) as our preferred combination of ingredients for our dry eye formulation to target multiple mechanistic events that are operating in the pathophysiology of DES. For this purpose, we designed a novel formulation that includes micronization of the active herbal ingredients through jet milling to reduce the particle size and used a combination of functional oils that help in improve absorption in the small intestine. In the current study, we demonstrate the protective effect of a novel multi-ingredient formulation containing lutein/zeaxanthin, curcumin, and vitamin D3 (LCD). The effect of the formulation is tested in a rat model with experimentally induced DES to investigate the role of LCD at a molecular level.

## 2. Results

### 2.1. LCD Formulation

The particle size of both active herbal ingredients as well as LCD final formulation was determined and we observed that micronization has a significant impact on particle size and reduced the size approximately ten times for all the components measured in our experiments (Table 1).

We measured the all the three active ingredients in the final formulation using HPLC assay using in-house established reference samples to each of the actives to ensure that the total content of the each of the ingredient in the final formulation is not less than 33.26% of total curcuminoids, 3.47% of lutein, 0.7% of zeaxanthin, and 930 IU of vitamin D3. The HPLC chromatograms of curcuminoids, lutein/zeaxanthin, and vitamin D3 were shown in Supplementary Figures S1–S3.

### 2.2. Serum Biochemical Profile

The serum biochemical parameters for overall health were measured and shown in Table 2. We did not see any abnormal changes for the parameters measured across the experimental groups.

### 2.3. Changes in Ophthalmologic Findings

**Figure 1:**
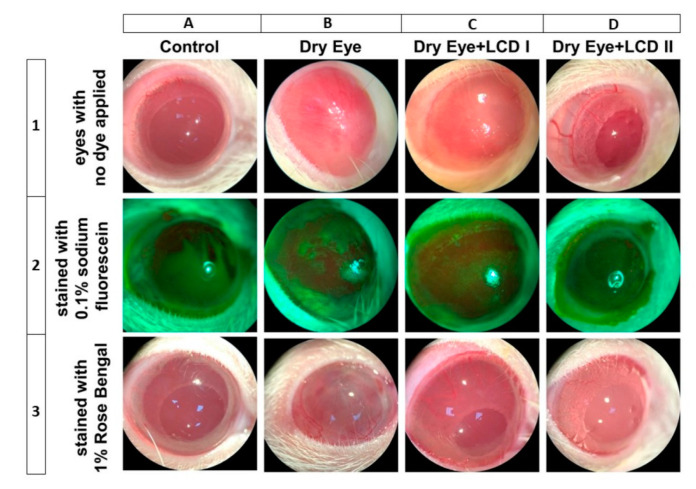
Effect of the combination of lutein/zeaxanthin, curcumin, and vitamin D (LCD) on Slit-lamp microscopic images of eyes in benzalkonium chloride (BAC) induced dry eye syndrome in rats. Control: No treatment; Dry Eye: BAC-induced dry eye syndrome; LCD I; Dry Eye + LCD I: BAC-induced dry eye syndrome plus LCD I (100 mg/kg body weight); Dry Eye + LCD II: BAC-induced dry eye syndrome plus LCD II (200 mg/kg body weight). Each representative vertical column contains the images of eyes from different groups (*n* = 7 per group); Horizontal rows respectively from up to down contain images of eyes with no dye applied (upper row, 1), stained with 0.1% sodium fluorescein (middle row, 2) and stained with 1% Rose Bengal (lower row, 3). A normal ocular surface of the rat in the control group is shown in A1. The cornea is transparent and the details of the iris and pupil can be seen behind the cornea. No sign of inflammation and pathologic staining with sodium fluorescein (A2) and Rose Bengal (A3) was observed on the ocular surface of rat eyes belongs to the control group. Slit-lamp microscopy shows us more than 2 mm ciliary hyperemia and vascularized cornea of rat induced by BAC (B1). Iris and pupil are invisible due to the scar and vascularization. Diffuse staining with plaques (B2) and confluent spots (B3) are observed in stained corneas by sodium fluorescein and Rose Bengal, respectively. The dose-dependent positive effect of LCD is seen in the images C1 and D1. Scar formation and vascularization of the cornea were recovered partly and almost fully in LCD I (C1) and LCD II (D1) groups, respectively. Iris and pupil details can be distinguished in C1 and more clearly seen in the D1. Similarly, lesser staining of the ocular surface was observed for both sodium fluorescein and Rose Bengal dye in LCD I (C2-3) and LCD II (D2-3) groups.

In the study, we investigated the effects of two different doses (100 or 200 mg/kg body weight) of the combination of lutein/zeaxanthin, curcumin, and vitamin D3 (LCD) in rats benzalkonium chloride (BAC)-induced dry eye syndrome. The effects of two doses of LCD on the alterations of the ocular surface are shown in Figure 1. Each vertical column from left to right represents each group (*n* = 7): control, dry eye, dry eye + LCD I (100 mg/kg), and dry eye + LCD II (200 mg/kg), respectively. Horizontal rows contain ophthalmic image samples belonging to each group. Images of eyes with no dye applied, stained with 0.1% sodium fluorescein, and stained with 1% Rose Bengal are placed from top to bottom, respectively. In the control group, there was no sign of ocular surface inflammation and staining (Vertical column A). Ocular surface damage induced by BAC is seen in vertical column B. Ciliary hyperemia was more than 2 mm and the iris and pupil were not visible behind the neovascularized cornea (B1). Severe staining with plaques was detected in the fluorescein test (B2). Rose Bengal staining revealed confluent spots on the cornea and conjunctiva (B3). The improvement due to LCD on the damaged ocular surface is shown in vertical columns C and D. Oral administration of LCD partially ameliorated ocular surface damage with LCD 2 (200 mg/kg) performing better than LCD 1 dose (100 mg/kg).

Ciliary hyperemia was reduced with better visibility of pupil and iris details in case of animal groups dosed with LCD 1 and LCD 2 as compared to dry eye group without treatment (C1 and D1). Similarly, lesser staining of the ocular surface was observed for both sodium fluorescein and Rose Bengal dye in treatment groups (C2-3 and D2-3).

**Figure 2:**
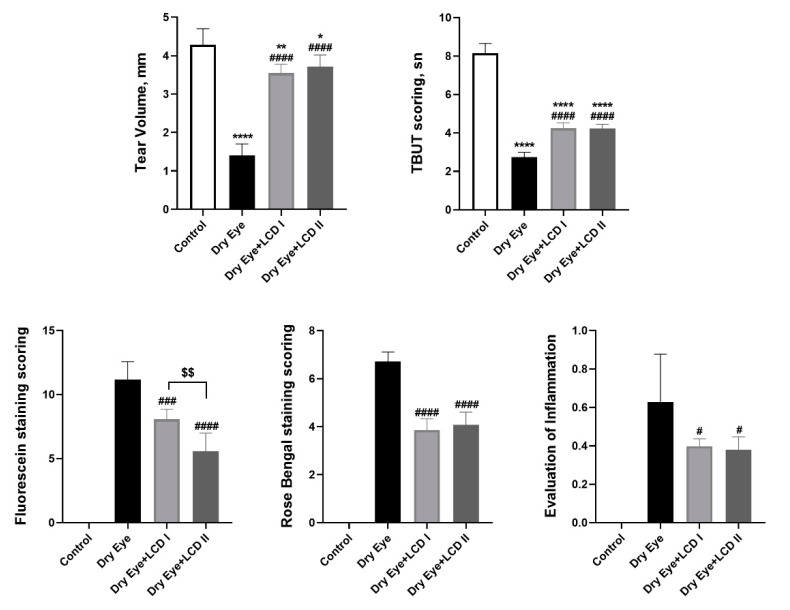
Effect of the combination of lutein/zeaxanthin, curcumin, and vitamin D (LCD) on tear volume, tear breakup time (TBUT) scoring, Fluorescein staining scoring, Rose Bengal staining scoring and evaluation of inflammation in benzalkonium chloride (BAC)-induced dry eye syndrome in rats (*n* = 7 per group). Control: No treatment; Dry Eye: BAC-induced dry eye syndrome; LCD I; Dry Eye + LCD I: BAC-induced dry eye syndrome plus LCD I (100 mg/kg body weight); Dry Eye + LCD II: BAC-induced dry eye syndrome plus LCD II (200 mg/kg body weight). Data are presented as a bar with means and standard deviations. (ANOVA and Tukey′s *post-hoc* test. Statistical significance between groups is shown by: * *p* < 0.05; ** *p* < 0.01; **** *p* < 0.0001 compared as control group; **^#^** *p* < 0.01; **^###^** *p* < 0.001; **^####^** *p* < 0.0001 compared as Dry Eye group; ^$$^ *p* < 0.01 compared as Dry Eye + LCD I group).

**Figure 3:**
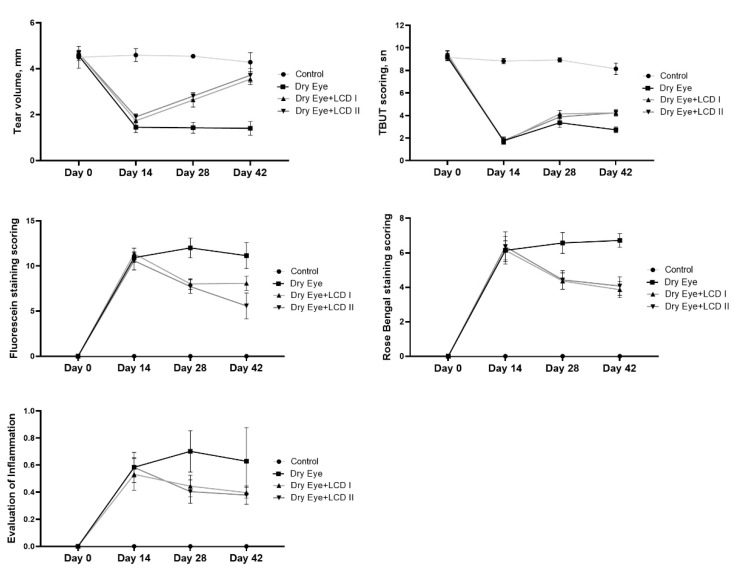
Effect of the combination of lutein/zeaxanthin, curcumin, and vitamin D (LCD) on tear volume, tear breakup time (TBUT) scoring, Fluorescein staining scoring, Rose Bengal staining scoring and evaluation of inflammation in benzalkonium chloride (BAC)-induced dry eye syndrome in rats on days 0, 14, 28 and 42 (*n* = 7 per group). Control: No treatment; Dry Eye: BAC-induced dry eye syndrome; LCD I; Dry Eye + LCD I: BAC-induced dry eye syndrome plus LCD I (100 mg/kg body weight); Dry Eye + LCD II: BAC-induced dry eye syndrome plus LCD II (200 mg/kg body weight).

Application of BAC on eyes resulted in symptoms of dry eye that included ophthalmic changes including tear volume and scoring for TBUT, fluorescein, and Rose Bengal staining and gross inflammation (*p* < 0.05) (Figure 2). However, treatment with LCD 1 & 2 significantly improved (*p* < 0.05) all the scores as mentioned above, and significantly reduced the fluorescein staining scores (*p* < 0.05). Graphs of time-dependent changes in parameters are presented in Figure 3. An improvement in the above readings with the addition of LCD was detected in the second week of the treatment and became more evident in the fourth week of treatment with maximum improvement observed in case of tear volume.

### 2.4. Changes in Oxidative Status

**Figure 4:**
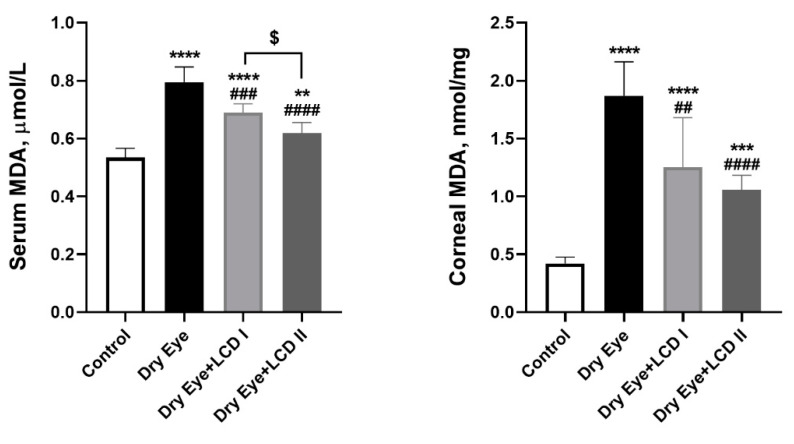

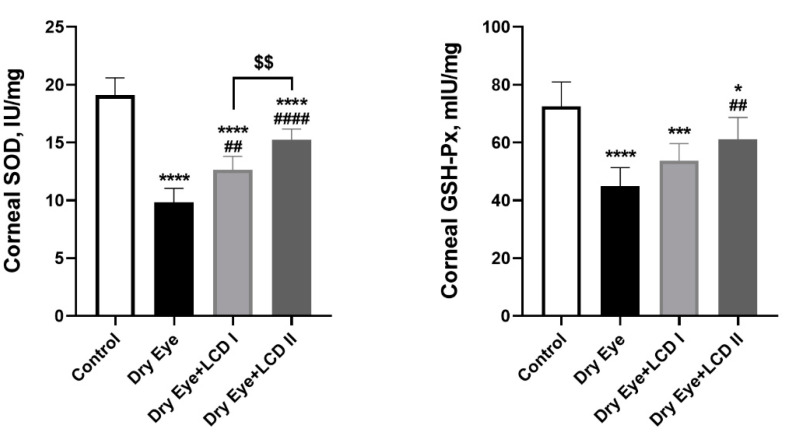
Effect of the combination of lutein/zeaxanthin, curcumin and vit D (LCD) on serum malondialdehyde (MDA), corneal MDA, corneal superoxide dismutase (SOD), and corneal glutathione peroxidase (GSH-Px), in benzalkonium chloride (BAC)-induced dry eye syndrome in rats (*n* = 7 per group). Control: No treatment; Dry Eye: BAC-induced dry eye syndrome; LCD I; Dry Eye + LCD I: BAC-induced dry eye syndrome plus LCD I (100 mg/kg body weight); Dry Eye + LCD II: BAC-induced dry eye syndrome plus LCD II (200 mg/kg body weight). Data are presented as a bar with means and standard deviations. (ANOVA and Tukey′s *post-hoc* test. Statistical significance between groups is shown by: * *p* < 0.05; ** *p* < 0.01; *** *p* < 0.001; **** *p* < 0.0001 compared as control group; **^##^** *p* < 0.01; **^###^** *p* < 0.001; **^####^** *p* < 0.0001 compared as Dry Eye group; ^$^ *p* < 0.05; ^$$^ *p* < 0.01 compared as Dry Eye + LCD I group).

Figure 4 shows the effects of BAC and LCD on corneal enzyme activities of SOD and GSH-Px and MDA levels in serum and the cornea. BAC induced oxidative stress in the ocular surface and increased serum & corneal levels of MDA, decreased corneal levels of SOD and GSH-Px, whereas LCD partially ameliorated the effect of oxidative stress induced by BAC (*p* < 0.05). The improvement was more obvious in the LCD 2 group compared with the LCD 1 group (*p* < 0.05).

### 2.5. Changes in Protein Levels

**Figure 5:**
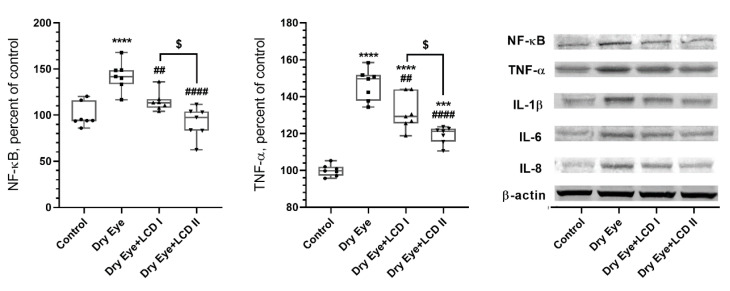

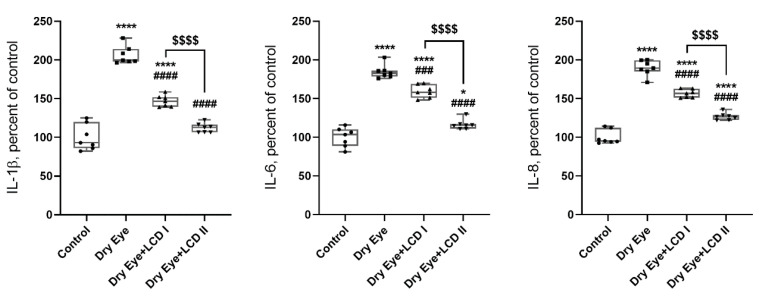
Effect of the combination of lutein/zeaxanthin, curcumin and vit D (LCD) on NF-κB, TNF-α, IL-1 β, IL-6, and IL-8 protein levels in benzalkonium chloride (BAC)-induced dry eye syndrome in rats (*n* = 7 per group). Control: No treatment; Dry Eye: BAC-induced dry eye syndrome; LCD I; Dry Eye + LCD I: BAC-induced dry eye syndrome plus LCD I (100 mg/kg body weight); Dry Eye + LCD II: BAC-induced dry eye syndrome plus LCD II (200 mg/kg body weight). Data are expressed as % of the control value. Western blot analysis (*n* = 3 at least) was performed with actin included to ensure equal protein loading. (ANOVA and Tukey′s *post-hoc* test. Statistical significance between groups is shown by: * *p* < 0.05; *** *p* < 0.001; **** *p* < 0.0001 compared as control group; **^##^** *p* < 0.01; **^###^** *p* < 0.001; **^####^** *p* < 0.0001 compared as Dry Eye group; ^$^ *p* < 0.05; ^$$$$^ *p* < 0.0001 compared as Dry Eye + LCD I group).

**Figure 6:**
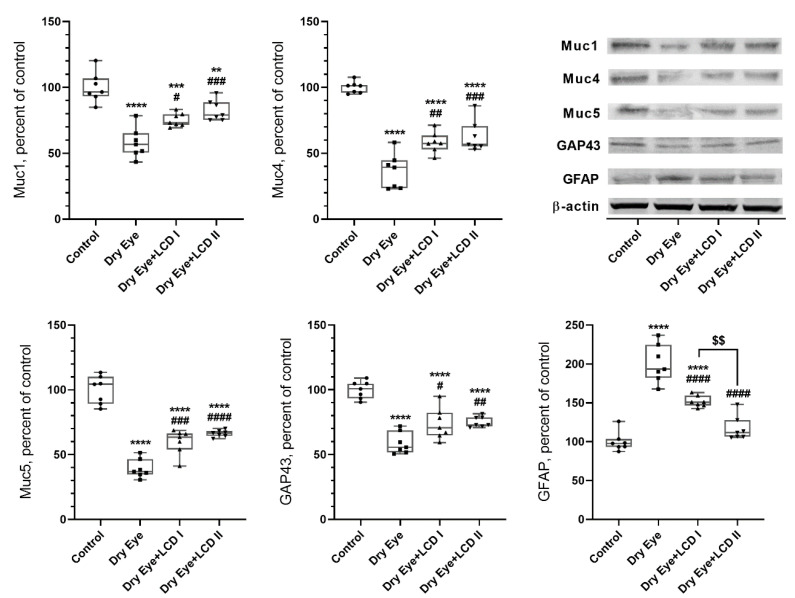
Effect of the combination of lutein/zeaxanthin, curcumin and vit D (LCD) on Muc1, Muc4, Muc5, growth-associated protein 43 (GAP43), and glial fibrillary acid protein (GFAP) benzalkonium chloride (BAC)-induced dry eye syndrome in rats. (*n* = 7 per group). Control: No treatment; Dry Eye: BAC-induced dry eye syndrome; LCD I; Dry Eye + LCD I: BAC-induced dry eye syndrome plus LCD I (100 mg/kg body weight); Dry Eye + LCD II: BAC-induced dry eye syndrome plus LCD II (200 mg/kg body weight). Data are expressed as percent of the control value. Western blot analysis (*n* = 3 at least) was performed with actin included to ensure equal protein loading. (ANOVA and Tukey′s *post-hoc* test. Statistical significance between groups is shown by: ** *p* < 0.01; *** *p* < 0.001; **** *p* < 0.0001 compared as control group; **^#^** *p* < 0.05; **^##^** *p* < 0.01; **^###^** *p* < 0.001; **^####^** *p* < 0.0001 compared as Dry Eye group; ^$$^ *p* < 0.01 compared as Dry Eye + LCD I group).

We measured the effect of BAC-induced DES on the levels of proteins in corneal tissue such as NF-κB, TNF-α, IL-1β, IL-6, IL-8, Muc1, Muc4, Muc5, GAP43, and GFAP (Figure 5 and Figure 6). BAC-induced eye symptoms are associated with a significant increase in levels of NF-κB, TNF-α, IL-1β, IL-6, IL-8, and GFAP and decrease in Muc1, Muc4, Muc5, GAP43 proteins (*p* < 0.001). However, LCD reversed the above protein expressions with LCD2 being more efficient than LCD1 (*p* < 0.05).

### 2.6. Changes in Histopathologic Findings

**Figure 7:**
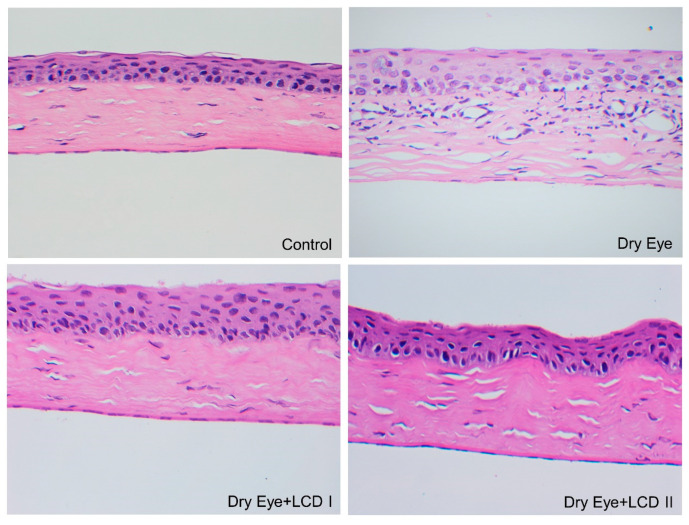
Effect of the combination of lutein/zeaxanthin, curcumin and vit D (LCD) on histopathological changes in benzalkonium chloride (BAC)-induced dry eye syndrome in rats. Control: No treatment; Dry Eye: BAC-induced dry eye syndrome; LCD I; Dry Eye + LCD I: BAC-induced dry eye syndrome plus LCD I (100 mg/kg body weight); Dry Eye + LCD II: BAC-induced dry eye syndrome plus LCD II (200 mg/kg body weight). Control: A histological appearance of a normal cornea. Dry Eye: The increased thickness of the corneal epithelium, inflammatory cell infiltration, and edema under the epithelium. Dry Eye + LCD I: Mild epithelial thickness, mild edema under the epithelium, and disappearance in infiltration. Dry Eye + LCD II: Normal appearance in epithelium thickness and very mild edema.

**Figure 8:**
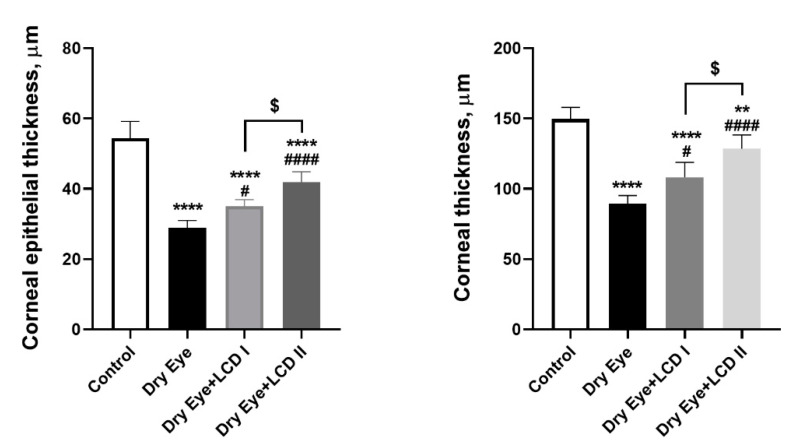
The effect of the combination of lutein, curcumin and vit D (LCD) on the corneal epithelial thickness and corneal thickness in benzalkonium chloride (BAC)-induced dry eye disease in rats (*n* = 7 per group). Control: No treatment; Dry Eye: BAC-induced dry eye syndrome; LCD I; Dry Eye + LCD I: BAC-induced dry eye syndrome plus LCD I (100 mg/kg body weight); Dry Eye + LCD II: BAC-induced dry eye syndrome plus LCD II (200 mg/kg body weight). Data are presented as a scatter plot with means and standard deviations. (ANOVA and Tukey’s *post-hoc* test. Statistical significance between groups is shown by: ** *p* < 0.01; **** *p* < 0.0001 compared as control group; **^#^** *p* < 0.05; **^####^** *p* < 0.0001 compared as Dry Eye group; ^$^ *p* < 0.05 compared as Dry Eye + LCD I group).

Representative images of rat corneas belonging to each group are shown in Figure 7. We observed that BAC-induced dry eye condition resulted in increased corneal epithelial thickness and inflammatory cell infiltration and edema under the epithelium were observed. LCD caused an improvement in the histopathologic findings in a dose-dependent fashion. The comparison of corneal epithelial thickness and corneal thickness profile is presented in Figure 8.

## 3. Discussion

In the current study, an optimized formulation for lutein/zeaxanthin and curcumin herbal ingredients and vitamin D3 developed by OmniActive Health Technologies (Mumbai, India) was used. As a part of the formulation process, we subjected turmeric powder (for curcumin) and marigold extracts (for lutein/zeaxanthin) to micronization which helped to reduce the particle size to <5 microns. The micronization process used here helps the formulation to reduce particle size and improve the dissolution rate, which in turn helps to enhance oral bioavailability [33,34]. We also used multiple functional oils as a part of the formulation, which is known to improve absorption in the gut [35,36,37]. BAC has been used successfully to develop in vivo dry eye disease models in mice and rabbits through topical applications into the eye [38,39,40]. We developed DES disease conditions in the rat by topical administration of BAC into the eye followed by oral administration of LCD formulation for 4 weeks. We observed that BAC induced significant damage to the ocular surface associated with pathophysiological features of DES as reported in the literature [41,42]. LCD formulation at both doses ameliorated the DES conditions with significant improvement in tear production, improved tear film stability, reduced corneal damage, and corneal surface inflammation which is further supported by histopathological findings. Further analysis of molecular markers indicates that LCD at both doses reduced oxidative stress and inflammation and markedly restored the levels of tear proteins such as mucins. We also observed that LCD provided a better protective effect at a dose of 200 mg/kg body weight as compared to 100 mg/kg body weight.

Although the etiology of DES in not clearly understood, existing animal and human studies validate the role of inflammation as one of the triggers to initiate the complex pathophysiology of DES [43,44,45,46]. An increase in pro-inflammatory cytokine levels in tear and corneal tissue has been correlated to poor tear function and increased tear osmolarity thus suggesting their role in underlying disease mechanism [47]. Inflammatory responses cause loss of goblet cells and glycocalyx mucin, and damage to epithelial cells, which further activate inflammation and impair tear film stability [8,48]. It has been established that MAPK and NF-κB signaling pathways play a critical role in the up-regulation of the inflammatory pathway and increases the levels of mediators including TNF-α, IL-1β, IL-6, IL-8 [8,49]. We found that LCD formulation at both doses significantly reduced overall inflammatory cell infiltration in corneal tissue and reduced levels of pro-inflammatory cytokines TNF-α, IL-1β, IL-6, IL-8 as well as transcriptional control protein NF-κB in corneal tissue.

Increased oxidative stress due to excess production of free radicals and reduced antioxidants causes cell damage and cell death. This has also been shown extensively in several conditions affecting the eye such as cataract, age-related macular degeneration, glaucoma, and DES [50]. Increased expression of xanthine oxidase, lipid peroxidation end products in the tear film and ocular surface, and decreasing expression of antioxidants such as SOD, catalase, and GSH-Px are reported in patients with DES [51,52,53]. Our data indicate that LCD formulation at both doses significantly reduced oxidative stress initiated by BAC-induced DES by restoring the levels of MDA and improving the levels of antioxidants SOD and GSH-Px.

GAP43 is a neuron-specific protein that regulates the regeneration and synaptic plasticity of neurons [54]. Expression of GAP43 on sensory axons in the cornea and alterations of expression in ocular surface disease has been demonstrated [55,56]. GFAP is an intermediate filament protein expressed in the central nervous system, mainly in astrocytes, and participates in cell-cell communication, maintaining cell shape, and repair after injury [57]. During HSV-1 infection in the cornea, a dynamic expression of GFAP was determined [58]. In the present study, we found that the levels of these two neural-associated proteins in the ocular surface of rats were depleted in BAC-induced DES were restored upon the administration of LCD formulation.

Mucins are glycocalyx proteins that play an important role in keeping the hydrophilicity of the tear film. It makes the tear film stable and reduces the surface tension, allowing uniform spreading of the aqueous layer over the surface of the eye. Muc1, Muc4, Muc5 are members of the mucin family, which are glycosylated phosphoproteins. Muc1 and Muc4 are transmembrane mucins, while Muc5 is gel-forming mucin located on the apical surface of epithelial cells. These mucins serve lubricative, protective, and cell- signaling functions involved in the NF-κB pathway [59,60,61]. The presence of mucin in conjunctival and corneal epithelial cells, goblet cells, lacrimal gland, and in tears has been reported [62,63]. Absence or reduced levels of mucins and alterations its distributions on the ocular surface are associated with ocular surface diseases such as conjunctivitis and DES. Kardon et al. showed that Muc1 null mice tended to develop blepharitis and conjunctivitis [64]. Danjo et al. detected an alteration of the distribution of mucin in patients with DES [65]. Furthermore, the association of reduced levels of Muc5AC and DES is reported in clinical and experimental studies [66,67,68,69]. In the current study, we found decreased levels of Muc1, Muc4, and Muc5 on the ocular surface of rats with DES which was significantly restored with LCD administration at both doses.

## 4. Materials and Methods

### 4.1. Preparation of LCD Formulation

All food-grade materials were used in the preparation and sourced as follows; standardized turmeric extract (95% total curcuminoids) from Synthite (Kolenchery, India), marigold flower extract prepared in-house by saponification and thermal isomerization reaction of marigold flower oleoresin, algal source of vitamin D3 (Avalon City, India), Medium Chain Triglyceride (MCT) oil and linseed oil (AAK Kamani, Mumbai, India), olive oil (Cargill India Pvt. Ltd., Haryana, India), sunflower lecithin from VAV lipids, (AAK Kamani, Mumbai, India), tocopherol from Matrix fine sciences, India and thyme oil (Sigma Aldrich, St. Louis MO, USA). Turmeric extract and marigold flower extract were micronized to reduce particle size (<5μ as measured by Malvern particle sizer 3000 according to the instruction from manufacturer) using jet mill micronizer (8” Pneumatic Bag Shaking model, Promas Engineers, Mumbai, India) with primary pressure of 6 bars and secondary pressure of 9 bars. 180 g of sunflower lecithin is dissolved in 3133 g of MCT oil at 70–80 °C with constant stirring followed by the addition of 9.63 g of vitamin D oil, 900 g of linseed oil, 450 g of thyme oil, and 180 g of tocopherol. The suspension is cooled down to 40 °C and 3031 g of micronized turmeric extract and 485 g of micronized marigold extracts were added with constant stirring to ensure the formation of a homogenized solution. Each of the active ingredients (lutein/zeaxanthin, curcuminoids, and vitamin D) in the preparation were analyzed by HPLC using reference standard to ensure each gram of the final product contains not less than 33.26% of total curcuminoids, 3.47% of lutein, 0.7% of zeaxanthin, and 930 IU of vitamin D3.

### 4.2. Particle Size Determination

We measured particle size for turmeric extract, marigold extract, and final formulation. The volume means diameter was measured using low angle laser light scattering (Malvern Masterizer 3000, Malvern Instruments LTD, Worcestershire, UK). The particles were suspended in deionized water containing 0.05% (*w*/*v*) Tween-80 and bath sonicated for 2 min, prior to measurements to ensure there was no aggregation. Particle size distribution (PSD) D90 in microns which is the portion of particles with diameters below a specific value is 90% is expressed as an average of 5 consecutive reading.

### 4.3. HPLC Assay for Active Ingredients

The samples were assayed using an Agilent 1200 HPLC System (Agilent Technologies, Santa Clara, CA, USA). For turmeric extract sample preparation 150 mg of turmeric was weighed, 15 mL of acidic buffer (pH 4.0) was added and sonicated for 5 min with intermediate shaking. After cooling, 50 mL tetrahydrofuran was added, the extract was sonicated for 5 min, and a volume of up to 100 mL was made with tetrahydrofuran (sample stock solution A). Next, the extract was pipetted with 2.0 mL of sample stock solution A into a 25 mL volumetric flask, diluted with the mobile phase and filtered through a 0.45-micron filter. Then, the sample was run with an HPLC system using the run condition shown in Table 3. Total curcuminoids were expressed as the sum of the curcumin (%) + desmethoxycurcumin (%) + bisdesmethoxy curcumin (%). For lutein and zeaxanthin sample preparation, 500 mg of marigold extract was weighed into a 100 mL volumetric flask, 70 mL of analytical grade ethyl acetate was added and the sample was shaken and sonicated for 10 min. After cooling, the sample was diluted to 100 mL with ethyl acetate and 5 mL of the above solution mixed and diluted to 25 mL with the mobile phase. Then, the sample was filtered the solution through a 0.45-micron filter, and clear filtrate was used for HPLC analysis under the run conditions as shown in Table 3. For vitamin D3 sample preparation, 1.5 g of sample was weighed and 30 mL of ethanol was added and mixed. Next, 5 mL of glycerin and 7 mL of 50% potassium hydroxide solution were added and the flask was refluxed for 2 h in a water bath maintained at 85–100 °C temperature. After 2 h, the flask was cooled and the contents transferred into a separator and extracted five minutes each time with 3 × 50 mL of ether. After completion of the ether extractions, the combined ether layer was washed with 5 × 50 mL of water or until alkali-free as measured by pH paper. Then, the ether layer was filtered through a funnel containing sodium sulfate was spread over a cotton bed into a dry 200 mL volumetric flask. The extract was evaporated using a steam bath, cooled and the residue dissolved in 100 mL of mobile phase by sonication for 2 min, mixed and filtered the final solution through a 0.45-micron filter, and the solution was injected into the column with the run conditions as shown in Table 3.

### 4.4. Animals and Experimental Design

Twenty-eight female Wistar rats (age: 8 weeks, weight: 180 ± 20 g) purchased from the Experimental Research Center of the University of Firat, Elazig, Turkey, were maintained in a controlled environment with a 12:12-h light-dark cycle at 22 °C throughout the study and given free access to rat chow and water. All experiments were conducted under the National Institutes of Health’s Guidelines for the Care and Use of Laboratory Animals in compliance with the Association for Research in Vision and Ophthalmology (ARVO) Statement for the Use of Animals in Ophthalmic and Vision Research and approved by the Ethics Committee of Firat University (Ethic code 2019/89-136).

Seven rats were selected randomly and kept aside as a control group and DES was induced in the remaining 21 rats through topical application of BAC solution (0.2%, Sigma-Aldrich, St. Louis, MO, USA) twice daily for 14 days. At the end of the second week, 21 rats with DES were randomly assigned into three groups: the dry eye group (*n* = 7), the dry eye + LCD 1 group (*n* = 7), and the dry eye + LCD 2 group (*n* = 7). 100 mg/kg and 200 mg/kg body weight of the LCD formulation was given to the Dry eye + LCD 1 and LCD 2 groups, respectively, by oral gavage for 4 weeks.

The ocular surface of all rats was evaluated by a masked ophthalmologist at baseline, second, fourth, and sixth weeks of the study under general anesthesia by intraperitoneal injection of 5% (*w*/*v*) chloral hydrate (0.7 mL/100 g body weight). The ocular surface was evaluated for tear volume, TBUT, corneal staining with fluorescein and Rose Bengal stains, and corneal inflammatory index scoring. At the end of the sixth week, animals in all groups were s-sacrificed by cervical dislocation under xylazine (10 mg/kg, i.m.) and ketamine (50 mg/kg, i.m.) anesthesia on the same day, and blood samples were collected for biochemical analysis. Cornea and orbital tissues were obtained for histological examination and protein expression analysis.

### 4.5. Tear Volume Measurement

The tear volume was measured using the modified Schirmer test with a strip of Whatman 41 filter paper in a similar fashion as done in humans [39]. Briefly, the process involved pull down the lower eyelid to visualize the palpebral conjunctiva and one end of the strip was placed on the palpebral conjunctiva, aligning the lateral one-third of the lower eyelid. The strip was held in place for 15 s and then the length of the wetted portion of the strip was measured in millimeters. For more accurate measurement, the test was repeated three times to obtain an average final score.

### 4.6. TBUT Scoring

Sodium fluorescein (1 µL) was applied to the conjunctival sac and the eyelids of anesthetized rats, were closed manually for the distribution of stain, and the cornea was examined using cobalt blue illumination of the slit-lamp microscope. The duration between the eyelids being kept open and the appearance of the first random dry spot on the cornea was counted and recorded in seconds. The test was repeated three times to obtain an average final score.

### 4.7. Fluorescein Staining Scoring

Following the application of sodium fluorescein into the conjunctival sac as above, the cornea was divided into four imaginary quadrants and examined for separate or confluent staining spots that indicate corneal epithelial damage using a slit-lamp microscope. The intensity of corneal epithelial damage was evaluated in each quadrant and graded as follows: 0 = no staining, 1 = slightly punctate staining (<30 spots), 2 = punctate staining (>30 spots, but not diffuse), 3 = severe diffuse staining, but not positive plaque, and 4 = positive fluorescein plaque. The sum of the scores of the four quadrants was considered the final score (the range of 0–16 points).

### 4.8. Rose Bengal Staining Scoring

The cornea and conjunctiva were viewed under a slit-lamp microscope for possible staining due to disruption in the protective mucin coating at 15 s following the installation of 1 µL of 1% Rose Bengal into the conjunctival sac. The previously described van Bjisterveld system was used to grade the extent and intensity of staining as; 1+ = few separated spots, 2+ = many separated spots, and 3+ = confluent spots using the range of 9 points [70].

### 4.9. Evaluation of Inflammation

Ciliary hyperemia and central and peripheral corneal edema were taken into consideration for grading inflammation. When performing ciliary hyperemia grading, the extent of hyperemia extending from the limbus to the periphery was considered (absent = 0, less than 1 mm = 1, between 1 and 2 mm = 2, more than 2 mm = 3). Central and peripheral corneal edema were graded respectively: none = 0, visible iris details = 1, no visible iris details = 2, no visible pupil = 3. The final score was the sum of the three parameters divided by nine.

### 4.10. Biochemical Analyses

To investigate the possible systemic adverse effects of LCD formulation, serum biochemical profile including, glucose, creatinine, blood urea nitrogen (BUN), total protein (TP), albumin (ALB), globulin (GLOB), and total bilirubin (TBIL) concentrations and activities of alanine aminotransferase (ALT) and aspartate aminotransferase (AST) were analyzed using a portable automated chemistry analyzer (Samsung LABGEO PT10V, Samsung Electronics Co., Suwon, Korea) following centrifugation of blood samples at 3000 g for 10 min. The activities of SOD and GSH-Px were determined using commercially available kits (Cayman Chemical, Ann Arbor, MI, USA) by the manufacturer’s instructions. MDA analyses, a high-performance liquid chromatography (HPLC) apparatus consisting of a UV–vis SPD-10 AVP detector, a CTO-10 AS VP column, and 30 mM KH2PO4 and methanol (82.5:17.5, *v*/*v*, pH 3.6) at a flow rate of 1.2 mL/min were used (Shimadzu, Kyoto, Japan). Column waste was monitored at 250 nm.

### 4.11. Histological Analyses

Eyes were fixed (4% paraformaldehyde and then paraffin) and sectioned into 5-µm slices using a microtome. Corneal and conjunctival tissues were stained with hematoxylin and eosin (H&E) and examined using light microscopy.

### 4.12. Western Blot Analyses

One cornea was rapidly removed from rats following sacrification. Seven rat corneas for each group were pooled and the total proteins were extracted in the cornea samples. For total protein extracts, 40 µg for each sample, were mixed with loading buffer (50 mmol/L Tris, pH 6.8, 150 mmol/L NaCl, 2% [*w*/*v*] SDS, 20% [*v*/*v*] glycerol, 5% [*v*/*v*], mercaptoethanol, 0.002% [*w*/*v*], bromophenol blue) and boiled for 5 min. Total corneal protein content was measured using a NanoDrop apparatus (MaestroGen, Las Vegas, NV, USA). Equal amounts of protein (20 μg) were electrophoresed and then transferred to nitrocellulose membranes (Schleicher and Schuell Inc., Keene, NH, USA). Nitrocellulose blots were washed in phosphate-buffered saline (PBS) for 5 min and blocked with 1% bovine serum albumin in PBS for one hour prior to administration of the primary antibody. The phosphorylated form of antibodies against Muc1, Muc4, Muc5, GAP43, GFAP, NF-κB, TNF-α, IL-1β, IL-6, and IL-8 (Abcam, Cambridge, UK) was diluted in a concentration of (1:1000) in a PBS buffer which contains 0.05% of tween 20. The secondary antibodies against these proteins were conjugated in the same buffer containing 0.05% Tween 20 (1:1000). All the primary and secondary antibodies were purchased from Abcam (Abcam). Monoclonal mouse antibody against β-actin (A5316; Sigma) was used as the loading control. Blots were performed at least three times to confirm the reproducibility of the results. The densitometric analyses of bands were detected with an image analysis system, Image J (National Institute of Health, Bethesda, MD, USA).

### 4.13. Statistical Analyses

The sample size of the study was determined to be 28 rats (*n* = 7; 4 groups) using the G*Power package program (Version 3.1.9.2) with alpha error 0.05 and 85% power with effect size 0.80, as calculated from previous studies [71,72]. The IBM Statistical Package for the Social Sciences statistical package (SPSS Statistics for Windows Version 22.0, 2013, IBM Corp., Armonk, NY, USA) was used to analyze the data. In this study, conformity to the assumption of normality from the prerequisites of the parametric tests was assessed using the Shapiro-Wilk test and the homogeneity of the variances was checked using the Levene test. Analysis of variance (ANOVA) was performed to determine the differences between the groups, and the *post-hoc* Tukey test was used for multiple comparisons of the groups. Data are presented as means and standard deviations for the groups. Statistical significance was accepted as *p* < 0.05.

## 5. Conclusions

We have developed a novel formulation that is beneficial in targeting molecular pathways of DES and ameliorates symptoms of the disease as demonstrated by the BAC-induced DES rat model. We also demonstrate our formulation to disrupt the disease mechanism by reducing the inflammatory response and oxidative stress and improve tear secretion and quality of tear by restoring the expression of neurotrophic factors and glycocalyx protein levels. We believe that our formulation with well-established and safe nutraceutical ingredients along with vitamin D developed as oral dosage form is a convenient and effective alternative to eye drops and existing pharmacological agents. These findings will be further validated in the near future when we conclude our ongoing human studies.

## Figures and Tables

**Table 1:** Particle size determination of particles.

	PSD(D90), in Microns		
	Turmeric Extract	Marigold Extract	LCD Oil Suspension
Before micronization	18.3 ± 1.9	17.5 ± 1.4	30.3 ± 2.5
After micronization	3.71 ± 0.4	2.12 ± 0.2	3.9 ± 0.3

**Table 2:** Effect of the combination of lutein/zeaxanthin, curcumin, and vitamin D (LCD) on serum biochemical parameters in benzalkonium chloride (BAC) induced dry eye syndrome in rats (*n* = 7).

Items	Groups				*p*
	Control	Dry Eye	Dry Eye + LCD I	Dry Eye + LCD II	
Glucose, mg/dL	120.43 ± 11.97	123.57 ± 7.32	122.86 ± 11.14	119.29 ± 7.72	0.826
Creatinine, mg/dL	0.39 ± 0.11	0.38 ± 0.09	0.40 ± 0.12	0.38 ± 0.08	0.974
BUN, mg/dL	23.24 ± 2.14	22.93 ± 0.91	22.54 ± 1.71	21.24 ± 2.82	0.289
TP, g/dL	7.03 ± 0.57	6.90 ± 0.50	6.60 ± 0.37	6.93 ± 0.34	0.352
ALB, g/dL	3.84 ± 0.39	3.66 ± 0.16	3.61 ± 0.21	3.73 ± 0.24	0.410
GLOB, g/dL	3.76 ± 0.35 ^a^	3.49 ± 0.22 ^ab^	3.19 ± 0.26 ^b^	3.36 ± 0.35 ^ab^	0.012
ALT, U/L	86.71 ± 7.54	83.00 ± 6.35	87.29 ± 10.06	84.29 ± 9.89	0.764
AST, U/L	121.00 ± 16.21	117.29 ± 13.31	122 ± 20.67	119.57 ± 12.03	0.950
TBIL, mg/dL	0.21 ± 0.03	0.22 ± 0.02	0.20 ± 0.02	0.24 ± 0.03	0.162

LCD: the combination of lutein/zeaxanthin, curcumin, and Vit D3; Control: No treatment; Dry Eye: Benzalkonium chloride (BAC) induced dry eye syndrome; LCD I; Dry Eye + LCD I: BAC-induced dry eye syndrome plus LCD I (100 mg/kg body weight); Dry Eye + LCD II: BAC-induced dry eye syndrome plus LCD II (200 mg/kg body weight). BUN: Blood Urea Nitrogen; TP: Total Protein; GLOB: Globulin; ALT: Alanine Aminotransferase; AST: Aspartate Aminotransferase; TBIL: Total Bilirubin; Data presented as mean and standard deviation (a,b). Means in the same line without a common superscript differ significantly (*p* < 0.05; ANOVA and Tukey′s *post-hoc* test). The assays were performed by Biochemical Analyzer.

**Table 3:** HPLC run condition for the samples.

	Curcuminoids	Lutein and Zeaxanthin	Vitamin D3
Column	Agilent Eclipse plus C18 (100 × 4.6) mm, 3.5 µm	Kromasil silica column (4 × 250) mm, 5 µm	Inertsil ODS C18 (250 × 4.6) mm, 5 μm
Wavelength	420 nm	446 nm	265 nm
Flow Rate	1.0 mL per minute	1 mL/minute	1 mL/min
Column oven Temp.	30 °C	25 °C	40 ± 2 °C
Sample oven temperature	25 °C	15 °C	20 °C
Injection volume	20 µL	20 µL	50 µL
Run time	15 min	20 min	20 min
Mobile phase	MP-A (Buffer): MP-B (THF) 600:400 ratio	hexane: ethyl acetate (500:500) *v*/*v*	600:400 (acetonitrile: methanol)
Diluent	Use the mobile phase	Mobile phase	Use the mobile phase

## Supplementary Materials

The following are available online at https://www.mdpi.com/1424-8247/13/10/295/s1. Figure S1: HPLC chromatograms of curcuminoids, Figure S2. HPLC Chromatograms of lutein and zeaxanthin, Figure S3. HPLC chromatograms of vitamin D3.
