# Supplementary material for: A Novel Integrated Active Herbal Formulation Ameliorates Dry Eye Syndrome by Inhibiting Inflammation and Oxidative Stress and Enhancing Glycosylated Phosphoproteins in Rats
Source: Pharmaceuticals (Basel). 2020 Oct 7;13(10):295. doi: 10.3390/ph13100295 (PMC7599565; doi:10.3390/ph13100295)
Supplement: Supplementary file 1 [file pharmaceuticals-13-00295-s001.pdf]

SUPPLEMENTARY FIGURES

A Novel Integrated Active Herbal Formulation Ameliorates Dry Eye Syndrome by Inhibiting Inflammation and Oxidative Stress and Enhancing Glycosylated Phosphoproteins in Rats

Omer Ersin Muz, Cemal Orhan, Fusun Erten, Mehmet Tuzcu, Ibrahim Hanifi Ozercan, Prafull Singh, Abhijeet Morde, Muralidhara Padigaru[5], Deshanie Rai[5], Kazim Sahin[2*]

```
Data file   : D:\CHEM32\DATA\2020\JULY\NG CURCUMIN\NGUS CURCUMIN ACCURACY->
Sample Name: STANDARD_WS/CUR/005                                           1
==========================================================================
Injection Date   : Sat, 11. Jul. 2020            Seq Line    :          2
Sample Name      : STANDARD_WS/CUR/005            Location    :     Vial 2
Acq Operator     : KINJAL                         Inj. No.    :          3
                                                  Inj. Vol.   :      20 µl

Acq. Method      : D:\Chem32\Data\2020\JULY\NG CURCUMIN\NGUS CURCUMIN  ->
Analysis Method  : D:\CHEM32\METHOD\CURCUMIN P.M\CURCUMIN P.M
```

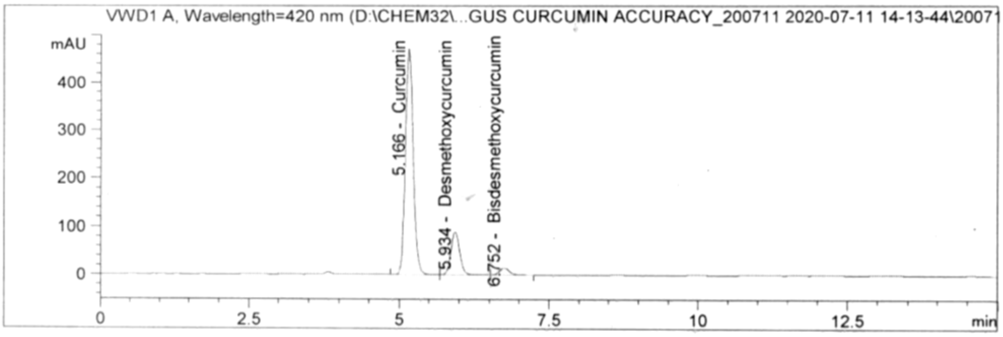

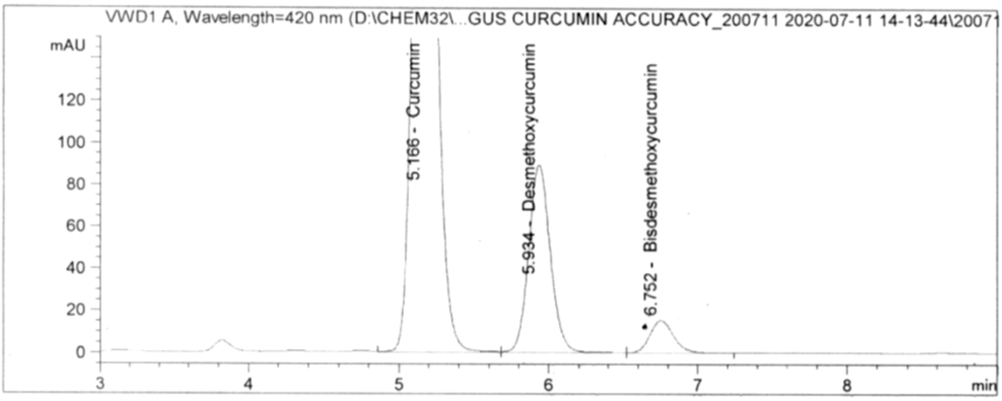

Signal 0: VWD1 A, Wavelength=420 nm

Peak #	Name	RT [min]	Area	Height	Area %	Symmetry
1	Curcumin	5.166	4315.830	470.371	80.089	0.863
2	Desmethoxycurcumin	5.934	903.635	89.261	16.769	0.883
3	Bisdesmethoxycurcumin	6.752	169.298	15.344	3.142	0.813

Figure S1. HPLC chromatograms of curcuminoids

```
Data file  : D:\CHEM32\DATA\2020\MAY\DRY EYES\DRY EYE LUTEIN_200514       1
             2020-05-14 11-31-51\2005140000004.D
===========================================================================
Injection Date & Time:   Thu, 14. May. 2020   12:37:48 PM
Sample Name       : STD WS/LZO/002
Acq Operator      : AP                              Seq Line    :3
Location          : Vial 2       Inj.Vol.:20 µl     Inj. No.    :2
Acq. Method       : D:\Chem32\Data\2020\MAY\DRY EYES\DRY EYE LUTEIN_200514
                    2020-05-14 11-31-51\DRY EYE  GRADIENT13.M
Analysis Method : D:\CHEM32\METHOD\DRY EYE  GRADIENT13.M
```

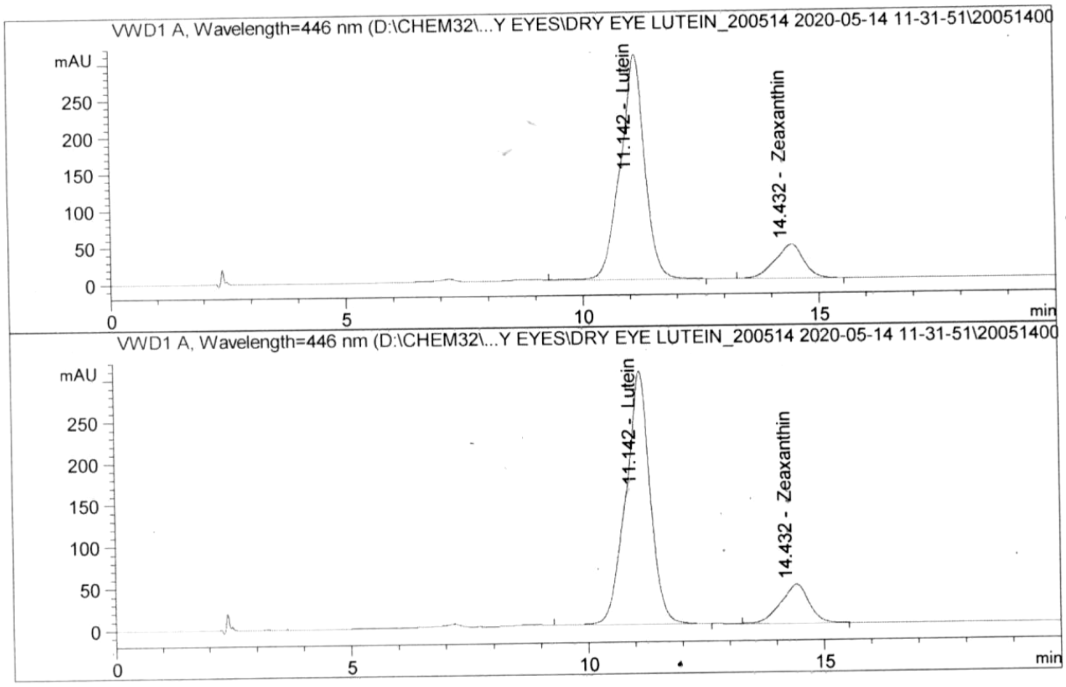

Signal 1: VWD1 A, Wavelength=446 nm

Peak #	Name	RT [min]	Area	Height	Height %	Area
1	Lutein	11.14	10772.57	303.89	86.86	85.07
2	Zeaxanthin	14.43	1890.74	45.99	13.14	14.93

Figure S2. HPLC Chromatograms of lutein and zeaxanthin

Area % Report

Sample Name: Standard Solution

Data File: C:\Enterprise\Projects\2020\JULY\RESULT\DRY EYE\Vitamin D3\20200716_Precision.rslt\002_Standard Solution-Rep1.dat

Method: C:\Enterprise\Projects\2020\Methods\Vitamin D3\STD 20MIN.met

Acquired: 16-07-2020 19:22:57 (GMT +05:30)

Printed: 17-07-2020 10:47:24 (GMT +05:30)

Vial No: 52 Injection Volume: 50

Data Description : {Data Description}

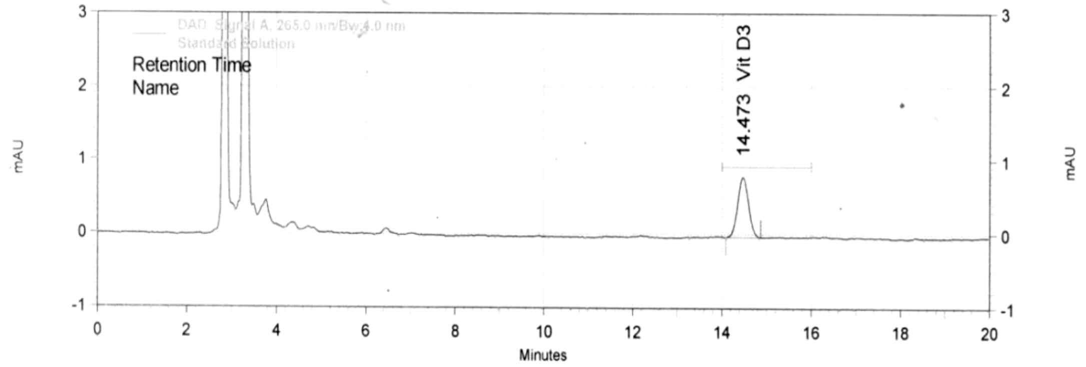

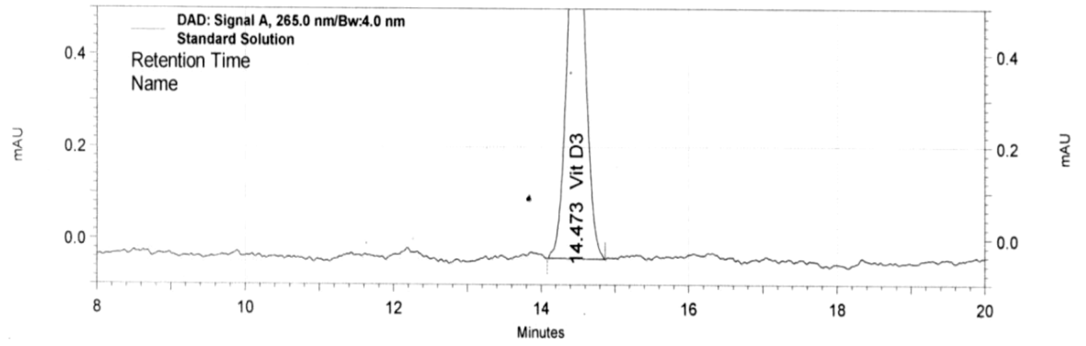

DAD: Signal A, 265.0 nm/Bw:4.0 nm Results

Name	Retention Time	Area	Theoretical plates (USP)	Asymmetry
Vit D3	14.47	1861126	16479	1.04
Totals		1861126		

Figure S3. HPLC Chromatograms of vitamin D3
